# Perceptions of drug users regarding Hepatitis C screening and care: a qualitative study

**DOI:** 10.1186/1477-7517-10-10

**Published:** 2013-06-20

**Authors:** Ashly E Jordan, Carmen L Masson, Pedro Mateu-Gelabert, Courtney McKnight, Nicole Pepper, Katie Bouche, Laura Guzman, Evan Kletter, Randy M Seewald, Don C Des-Jarlais, James L Sorensen, David C Perlman

**Affiliations:** 1 Beth Israel Medical Center, 120 East 16th St, Floor 12, New York, NY, 10003, USA; 2 Department of Psychiatry, San Francisco General Hospital, University of California, San Francisco, 1001 Potrero Avenue, Building 20, Suite 2100, San Francisco, CA, 94110, USA; 3 Center for Drug Use and HIV Research, 120 East 16th St, Floor 12, New York, NY, 10003, USA; 4 National Development and Research Institutes Inc, 71 West 23rd St. Floor 8, New York, NY, 10010, USA; 5 Prevention Point, San Francisco AIDS Foundation, HIV Prevention Project, San Francisco AIDS Foundation, 1035 Market Street, Suite 400, San Francisco, CA, 94103, California; 6 Mission Neighborhood Resource Center, 165 Capp Street, San Francisco, CA, 94110, California; 7 BAART Programs, 433 Turk Street, San Francisco, CA, 94102, California

**Keywords:** Hepatitis C, Illicit drug use, Injection drug use, Health care access

## Abstract

**Background:**

Illicit drug users have a high prevalence of HCV and represent the majority of newly infected persons in the U.S. Despite the availability of effective HCV treatment, few drug users have been evaluated or treated for HCV. Racial and ethnic minorities have a higher incidence and prevalence of HCV and higher HCV-related mortality. Factors contributing to poor engagement in care are incompletely understood.

**Methods:**

Fourteen mixed-gender focus groups of either African American or Latino/a drug users (N = 95) discussed barriers to HCV testing and treatment. Themes were identified through content analysis of focus group discussions.

**Results:**

Many drug users were tested for HCV in settings where they were receiving care. Outside of these settings, most were unaware of voluntary test sites. After testing HCV positive, drug users reported not receiving clear messages regarding the meaning of a positive HCV test, the impact of HCV infection, or appropriate next steps including HCV clinical evaluations. Many drug users perceived treatment as unimportant because they lacked symptoms, healthcare providers minimized the severity of the diagnosis, or providers did not recommend treatment. Mistrust of the motivations of healthcare providers was cited as a barrier to pursuing treatment. Social networks or social interactions were a source of HCV-related information and were influential in shaping drug users perceptions of treatment and its utility.

**Conclusion:**

Drug users perceived a paucity of settings for self-initiated HCV testing and poor provider-patient communication at test sites and during medical encounters. Notably, drug users reported having an unclear understanding about the meaning of a positive HCV test, the health implications of HCV infection, the importance of clinical evaluations and monitoring, and of treatment options for HCV. Efforts to improve the delivery of clinical messages about HCV infection for drug users at test settings and clinical encounters are needed.

## Background

Hepatitis C virus (HCV) is a blood-borne infection most efficiently spread via direct parenteral exposure through non-sterile injection practices [1-4]. The World Health Organization estimates a global prevalence of HCV of 2%, or 123 million people [5] most of whom are chronically infected. HCV is the most common chronic blood borne infection in the United States and worldwide, and accounts for roughly one quarter of all cases of cirrhosis and hepatocelluar carcinoma [6,7]. HCV is hyperendemic among people who inject drugs, representing the largest group of infected persons both worldwide, and in each country where HCV prevalence and risk factor data are available [8-10]. The estimated global prevalence of HCV among IDUs ranges from 9.8% to upwards of 97%, with most estimates falling between 50-90% in regions with long-standing endemic injection drug use [1,4]. The incidence of HCV among IDUs ranges regionally from 10 to 40 per 100 person-years at risk [1,11]. HCV is also transmitted sexually among men who have sex with men, often in association with non-injection illicit drug use [12,13].

HCV causes chronic infection with persistent viremia in the majority of those infected (~85%) [14]. As a result, chronically infected persons constitute a significant reservoir of HCV creating an environmental transmission dynamic that increases the probability that a non-sterile injection episode will be with a chronically HCV-infected person [1,11]. Important sequelae of chronic HCV are liver fibrosis leading to cirrhosis; liver failure; and hepatocellular carcinoma [15]. Studies suggest that over the course of two decades, 20-30% of chronically HCV infected persons will develop cirrhosis, with an estimated 10,000-20,000 early deaths [14]. In the United States, the disease burden is predicted to increase up to 3-fold over the course of the next 10–20 years [15]. The efficacy of HCV treatment has improved in recent years with the introduction of direct-acting antivirals (e.g., telaprevir and boceprevir) and the prospect of interferon-free regimens [16,17]. For many, fear of adverse effects of HCV treatment is a barrier to treatment initiation and may contribute to treatment non-adherence and treatment discontinuation [18-21]. While HCV treatment has the potential to cure the virus in 40-80% of patients, current treatment is arduous, lengthy, expensive and remains inaccessible for many drug users [15].

The majority of drug users remain out of HCV care, and few are engaged in treatment [18,21-24]. Many HCV positive drug users have not been evaluated for HCV treatment; are less likely to see an HCV specialist or to get an HCV RNA polymerase chain reaction (PCR) test to document chronic active infection; and are less likely to be receiving antiviral treatment for HCV compared to non-injection drug users [18,21,25]. Active drug use has been shown to not have a direct, negative effect on treatment efficacy [8,9,26-28]. It is estimated that less than half of drug users with chronic HCV have been offered treatment ever [17].

Racial/ethnic minorities are less likely to receive anti-retroviral therapy for HIV [29,30]. Some prior qualitative studies have highlighted drug users’ misconceptions and lack of understanding about HCV, racial and ethnic minorities have a higher incidence and prevalence of HCV and higher HCV-related mortality [18,31-35]. Drug users often have limited access to health care and may experience or perceive stigmatization that poses a barrier to care [23,35]. Additionally, some drug users report that drug use-related stigma is a barrier to HCV testing. Rates of HCV are higher in racial/ethnic minority drug users [36]. Further data to inform the delivery of clinical messages about HCV infection for drug users at test settings and clinical encounters are needed.

This study sought to explore racial/ethnic minority drug users’ attitudes, perceptions, and experiences regarding HCV and HIV testing, referrals and treatment, through focus groups with drug users in San Francisco and New York City. This paper presents data regarding HCV testing and care.

## Methods

### Study participants

Fourteen focus groups with a total of 95 participants were conducted in New York City (6 focus groups) and San Francisco (8 focus groups) in three recruitment settings: HIV primary care clinics, methadone maintenance treatment (MMT) programs and syringe exchange programs (SEP). During the course of the study, the HIV clinics both conducted HCV testing and the site in NYC provided on-site HCV treatment; the MMT programs offered anti-HCV testing, but neither viral load testing nor HCV treatment; and the SEPs did routinely offer HCV testing but offered no on-site HCV care. Eligibility criteria required that participants be 18 years of age or older; self-identify as African-American or Latino/a; and be receiving services at one of the recruitment sites. Participants were excluded from the study if they had severe cognitive impairment, suicidal ideation, or active psychosis. The study included persons who have used illicit drugs in the past 12 months by either injection or non-injection routes; non-injection illicit drug users were included because of data demonstrating rates of HIV in non-injectors comparable to injectors in many cities [3] and because of concerns of HCV transmission via drug using paraphernalia and networks [1]. The terms ‘drug users’ and ‘injection drug users’ (IDUs) are used throughout the text where appropriate. This manuscript reports on findings with respect to HCV testing and treatment. This study was approved by the Institutional Review Boards of Beth Israel Medical Center and the University of California, San Francisco.

Participants were recruited through staff referrals at each of the recruitment sites regardless of HCV status or prior testing experience. Participants were told that the goals of the focus group were to explore participants’ experiences with HIV and HCV testing and care. The number of participants in each group ranged from 3 to 12. All focus groups were of homogenous race/ethnicity, consisting of either Latino/a or African American participants. The rationale for race/ethnicity specific focus groups was to identify possible race/ethnicity specific issues with regard to HIV and HCV testing and care. All focus groups were conducted in English. Participants provided informed consent and were reimbursed $25 for their participation in the study.

Focus groups were conducted by PhD-level qualitative researchers, bi-lingual in English and Spanish; each group lasted roughly 90 minutes. The focus groups used a semi-structured qualitative interview guide designed to explore, in-depth, the following specific thematic areas related to HIV and HCV including: self-perceived risk; general knowledge of the viruses; prior experiences and current feeling about seeking testing; prior pre- and post-test experiences; and prior experiences accessing or remaining engaged in treatment. Further, the interview guide also included open ended queries about individual’s drug use histories, knowledge of their own HIV/HCV status, and perceptions about race/ethnicity in relation to testing and care (the focus group guide is available from the corresponding author). Participants recruited for these focus groups were not tested serologically: those recruited from HIV clinics were known to be HIV infected; for others HIV status was by self-report; and for all, HCV status was self-reported. 39% reported HIV infection (21% of the total reported HCV/HIV co-infection), 36% reported HCV mono-infection, and the rest reported unknown status. All focus groups were audio taped and transcribed verbatim.

### Qualitative data analysis

Transcripts were coded and analyzed using Atlas.ti V.5 software. At least two researchers individually reviewed and independently coded all transcripts and discussed ambiguities. Grounded theory [37] analytic techniques were used to seek patterns in the data and to develop emergent hypotheses about them. Analysis began by coding verbatim references containing any of the following codes: HCV/HIV testing, access to HCV/HIV care, HCV/HIV treatment, racial/ethnic minority status, co-infection and drug user status. Two emerging codes were added during the analysis: “medical mistrust” and “stigma”.

## Results

Fourteen focus groups were conducted, 6 in NYC and 8 in San Francisco. The 6 in NYC included one with Latino/a participants and one with African American participants at each of the three recruitment settings (MMT, SEP, and HIV clinic) with a total of 51 participants. The 8 in San Francisco included 2 with African American and 1 with Latino in MMT; 2 Latino and 1 African American at HIV primary care; and 1 African American and 1 Latino at SEP, with a total of 44 participants. The total sample of 95 participants was 41% female (n=39); average age was 45 years (minimum 32, maximum 58). The analysis discusses results related to access to HCV testing, post-test counseling and medical care, experience with HCV treatment and perceptions of HCV treatment. No differences between testing experiences emerged by gender or between focus groups in NYC and San Francisco hence, results are reported in aggregate.

### HCV testing

In focus groups, nearly all participants reported having been tested for HCV. Participants generally described an HCV testing experience that consisted of testing at the structured settings in which they were receiving care, with tests commonly having been initiated by health care providers with knowledge of participants’ risk factors for HCV. The primary settings in which participants were tested for HCV were MMTs and SEPs. Common to those who were or had been in MMT was a perception that routine HCV testing was a mandatory component of the intake exam and annual physicals for all MMT patients: “You’re on methadone, it’s a requirement anyway, to get tested for [HCV]” (African American male); no one reported objecting to being tested for HCV in this way. This perceived routinization of HCV testing was also reported by participants who underwent testing at health care sites where tests were usually initiated by health care providers and where the reason for the appointment was to receive care for other illnesses: “I did [HCV] testing when getting [treatment for] pneumonia” (Latino). Such testing often took place without participants being aware that they were tested for HCV: “I found out afterwards [that I was tested for HCV]. [The doctor] tested it on his own”. (Latino).

While testing for HCV was common among focus group participants, most reported being unaware of voluntary testing sites. Most participants were eager to have access to voluntary HCV testing. Focus group participants did not report seeking self-initiated HCV testing outside of MMT, SEP, jail and HIV primary care settings: “Most people just don’t know where to do it [HCV test], unless you go to the exchange and they happen to be doing it there” (African American male); “You have to find a way to get it [HCV test]; It ain’t like-come and get a hep C test. It’s like a best-kept secret” (African American male).

Underscoring the reality that HCV is a widely asymptomatic disease, only one participant reported seeking medical attention because they experienced symptoms associated with an HCV infection: “I had yellow jaundice. Like my urine was orange, real dark orange […] At least the doctor told me [I was HCV positive]” (African American female).

These patterns of HCV testing experiences contrasted with participant reports regarding HIV testing, which were characterized by frequent and self-initiated testing with access to ubiquitous testing sites: “HIV testing is much more accessible to me, more accessible than hepatitis C” (African American female). Participants had a high degree of awareness of available HIV testing sites: “The fact that they’re so accessible, I feel like if any day I feel like getting up and going to get [an HIV] test, I can get it the very same day” (Latina). Many participants reported self-initiated HIV testing every three to six months.

### Experiences with HCV post-test counseling and referrals

Despite their individual histories of drug use, many participants were surprised when they were first diagnosed with HCV: “My doctor took blood, and he tested it and he told me I had hepatitis C, and that was my first time knowing about it […] I was an injector”. (African American male)

Many participants found to be HCV positive reported receiving their results but coming away from post-test counseling without a clear understanding of the significance of the diagnosis or what next steps to take: “I found out I was hep C positive. [The doctor] told me the basics but they never really told me what the next step was”. (Latino) Participants reported confusion and uncertainty given their new situation: They had been given a diagnosis of HCV, but did not come away with a clear understanding of the health implications or what to do next:

You’re hep C positive, but now what? they should have a place to send them or I should have somebody some place at my facility to at least counsel them. Nobody has even spoken to them. (African American male)

They won’t refer you to nobody, see they just told me and just left me hanging. Just left me there. (African American female)

They didn’t give me nothing to go on. I had nothing to take home with me and sit down and study and go over myself… they don’t have nothing for the poor person that has contracted hep C. Nobody where I got tested at gave me any literature. (African American female)

Accompanying the feelings of uncertainty regarding an HCV diagnosis, many participants described feeling fatalistic with a generalized nihilism about managing their infection: “I don’t know what to do. Except just walk around dying from it” (African American male).

Participants specifically described a lack of explanation and clarity regarding the treatment options for HCV and they were eager for more information and a better understanding of HCV treatment: “When I first found out I had hepatitis C, they didn't suggest any kind of treatment. It was only two or three years later that they made me an appointment for the hospital to go” (Latina).

As part of this uncertainty about treatment, many people came away with an implicit message that there was not much else that could or needed to be done to treat HCV or to prevent liver damage. As one participant explained: “Everybody says there’s really nothing too much to do when you got that [HCV]. They just say, yeah, I got it, as far as hep C, and they [health care providers] just let you know” (African American male). Participants also reported disengaging from care once they found out they weren’t eligible for treatment or that treatment wasn’t necessary for them at that point in time.

So he told me that if you want, take a biopsy if you want it, that was my option. So I didn't do it. He said, but your liver seems like it's okay. The numbers are in a good-good place […] So I dropped it at that. (Latino)

Some participants, despite having been told they were HCV positive, did not believe they were infected because their providers did not offer them treatment: “I don't believe them [the doctors] for the simple fact they didn't give me, they didn't give me no medicine for it [HCV]”; “I’m sure he will if I have hep C, he will tell me take this and this medication. He will order it. So I do not believe I have hep C” (African American male).

### Experiences with HCV treatment evaluations

Among participants who reported receiving HCV treatment evaluations, many said that they were told by health care providers that due to the healthy state of their liver and the results of various tests to assess their infection, treatment was not recommended at that time. Some understood that treatment was not offered because there was no evidence of liver damage. Many participants reported not initiating HCV treatment because their providers either did not discuss or recommend it: “My doctor told the same thing that everything was fine, not to worry about it [HCV] […] that the numbers were low and that I didn't need no medication or anything” (Latina); “[My doctor told me] I didn’t need a treatment because it wasn’t bad […] My liver wasn’t inflamed, and I was doing okay […] there was no need for medication until years down the line” (African American male).

While some people came away from HCV evaluations understanding that treatment was indicated if there was substantial liver damage and may not be otherwise necessary, many participants did not have a thorough understanding and felt as if they were left in limbo with a positive diagnosis without clear options. Participants who were evaluated but not offered treatment commonly reported being counseled about reducing drug and alcohol use and avoiding excess acetaminophen, “[the doctor] told me that […] just don't drink any alcohol and don't abuse them, stuff that's going to irritate the liver”. (Latino)

There were few participants who reported being encouraged to have regular medical follow-up to monitor their HCV infection but without recommendation for treatment.

I went according to my doctor. He said that my viral load was okay, so I took—I took it like that, okay, so then I'm fine. Every month, you go see that doctor […] on a monthly basis and stay—stay with blood work. (Latino)

Few participants in the focus groups had initiated HCV treatment; however, of those who reported initiating treatment, they all discontinued treatment due to adverse drug reactions.

I decided to treat it [HCV] […] I only did it for like four months because I ended up getting some side effects […] The medication was doing things that I dislike […] I told [my doctor] that I am not taking it anymore. (Latino)

For the participants who reported being offered treatment, many reported that the low odds of eradicating the virus deterred them from initiating treatment. Additionally, one participant reported that their provider did not recommend treatment saying the patient was infected with an HCV genotype that was poorly responsive to treatment.

### Perceptions of HCV treatment

Knowledge and perceptions about HCV treatment often came from peers, and the messages communicated were often discouraging of treatment. While a few patients had previously initiated HCV treatment, most had no direct treatment experience. Participants reported that these communications with peers raised anxiety about the potential adverse side effects of the medication: “I didn’t even know what the process was […] I found out through someone who had hep C, and her experience through it” (Latino).

Participants with HCV were uniformly eager to learn more about HCV treatment and how to stay healthy. Among participants who reported discussing treatment with a health care provider, or who were offered treatment, the majority felt dissuaded from pursuing it. While some participants reported an interest in treatment, the consideration of “everything that goes with it”, including not wanting to endure the serious side effects of treatment, was the primary reason that participants chose not to initiate treatment. Among HCV-positive individuals in particular, there was a common perception that HCV treatment was worse than the disease due to the difficulty in coping with the length of treatment and medication side effects: “[There are] bad reactions that a lot of people have with medication. Some people get suicidal, depression […] They’d get lonely, you know, depressed, big, big stay of depression” (Latino).

Many participants while willing and even eager to consider HCV treatment, many articulated that these fears of lengthy treatment and severe adverse effects- discouraged them from pursuing treatment. Additionally, participants reported believing that treatment might be harmful to their liver, might cause HCV infection or other harmful physical adverse effects, and be inefficacious: “Interferon I’ve heard is the treatment for it but I’ve heard that the treatment is worse than the disease and it’s not effective”. (Latino) Such concerns lead many infected but asymptomatic participants to not seek treatment; as one participant explained it: “if it ain’t broke, don’t fix it” (African American male). This attitude was common; participants reported believing that it was more advantageous to their health to not seek treatment rather than pursue treatment and risk making their health worse: “my liver function is still good, so I’m not going [to] take something that’s going [to] make me worse” (Latino).

In contrast, across focus groups, participants’ overall knowledge about HIV and treatment options was extensive, regardless of their HIV status. Participants understood opportunistic infections; various tests indicating HIV/AIDS status; treatment outcomes; and the necessity of treatment to control the infection “If you’re told you got HIV […] it’s not like before, like it’s more manageable, you can live longer […] there’s so many drugs that can help with it”. (Latina)

In addition to their individual concerns about HCV, participants also regarded HCV as a virus infecting and affecting drug users rather than non-drug users. They concluded that the paucity of HCV services and the lack of effective treatment options were the result of stigma and marginalization of IDUs. In addition, participants described their belief that socioeconomic factors and insurance availability influenced their doctors’ decision making regarding the provision of HCV treatment.

If you have private insurance […] They’ll give you all the treatment and health that you want…but because they know that Medicaid is not going to pay them their money on time- they’re going to get paid […] they’re not ready and willing to offer this treatment [to those with Medicaid]. (Latino)

I think a lot has to do with – people who – the powers that be don’t use drugs like we use drugs. It [HCV] don’t affect them. (African American male)

One participant explained that she felt mistreated by her doctor: “They kick you to the side”. (African American female)

Mistrust of health care providers’ motivations manifested in other ways. Some participants reported believing that their providers were diagnosing, and even misdiagnosing HCV to receive insurance payments for their visits.

### Implications of drug use

One of the barriers participants reported complicating engagement in HCV care was active drug use. Participants reported that when they were using actively they were less likely to get tested for HCV, “Personally, I wouldn’t put down no syringe […] to go get tested [for HCV]” (Latino); “It took me so long [to get tested] because I was getting high”. (Latino) One participant explained that her commencing HCV care occurred “fast as my addiction would let me go”. (African American female) Active drug use not only emerged as a barrier to participants’ willingness to engage in HCV testing, but also as a barrier to clinical follow-up after receiving a HCV diagnosis. Participants reported that the consuming nature of drug use precluded any motivation to seek care. One participant attributed his active heroin use to his inability to schedule an appointment for an HCV evaluation from a referral he received after being tested for HCV:

Yes, [the doctor] told me … numerous times. I just didn’t do it. Either I forgot about it or was just too lazy to get up off my ass and go do it. I got a thing about keeping appointments […] it’s the dope’s fault. (African American male)

## Discussion

It is estimated that about 60-90% of drug users are infected with HCV [14,38,39]. Focus groups with drug users in MMT, SEP and HIV Primary Care reveal that prior to a diagnosis of HCV, most participants had a poor understanding of HCV and its significance. After being diagnosed, many participants reported not receiving a clear message regarding what the infection meant; their HCV status; and next steps, including follow-up evaluations and the availability, role and efficacy of treatment options. Participants also reported some mistrust of health care providers, recognizing that active drug use is a barrier and commonly reported not receiving referral for HCV clinical evaluation after receiving a positive test result.

Many drug users come into contact with drug treatment programs and/or drug related services such as needle exchange. These programs serve as important points of access for health services. In focus groups discussions, participants explained that programs for drug use served as primary settings in which they received HCV testing. SEPs and MMTs along with other clinical settings were structured settings in which participants reported receiving HCV testing. Our findings underscore the importance and utility of providing HCV testing in drug treatment programs or programs aimed at serving drug users. In our sample the majority of drug users had reported receiving at least one HCV test in the past; this has not been the case in all previous studies [35,39]. This may relate to participants’ having been recruited in clinical or harm reduction settings.

In contrast to ready access to voluntary HIV testing, participants in our study reported limited access to voluntary HCV testing. Participants in numerous studies reported feeling most comfortable accessing HCV testing and HCV-related services at sites where providers had an understanding of addiction and were accustomed to and respectful of drug users [15,40]. In our study, participant comments also suggested that in the health care systems they accessed, there were few settings available for voluntary HCV setting (e.g., mobile HIV testing but no HCV testing vans). In this way, and in the learned experience of our focus group participants, offering both targeted and voluntary testing at sites where drug users are already receiving services, in settings widely populated by drug users, could serve as effective points of entry for drug users to initiate and maintain care for their HCV infections.

Overall, participants reported a gap between testing and receiving referrals to medical evaluations following a positive HCV test result. Many publications document low rates of referral after testing positive [27,34,35] but it is usually assumed that this gap is due to patient non-adherence; our data demonstrates drug users perceive not having had received referrals. Participants reported feeling abandoned by clinicians, a finding that is consistent with other studies of HCV testing among drug users [15,41]. It is important to note that the same barriers that participants identified may also contribute to provider reluctance to initiative HCV treatment for drug users, however, several studies have highlighted that with appropriate attention to these issues, active drug users can be successfully treated for HCV [9].

Most participants in our study were unclear about how HCV infection should be evaluated and monitored, and about treatment. Some participants also perceived HCV treatment as something available to the wealthy and not to marginalized groups or those on Medicaid. Others were suspicious that HCV was diagnosed and treatment offered more for profit than to improve the health and well-being of patients. It is difficult to know whether these perceptions were based on a lack of understanding of HCV infection and treatment (a knowledge deficit) versus based primarily on emotional factors such as medical mistrust. The former would be amendable to educational efforts while the latter would require being addressed by other intervention strategies.

The fact that focus group participants reported relying heavily on peers for HCV treatment knowledge suggests that support from peers may be a valuable way to engage drug users in HCV care. This finding is consistent with qualitative studies that have been published on this topic [15,40]. This peer-gained knowledge of HCV and its treatment is maintained through peer relationships. These forums serve a critical role in disseminating information about HCV to those either untreated, out of care or who have not received adequate information from their providers [8].

The findings presented in this paper should be interpreted with some caution as reported experiences may not be generalizable to all drug users in all settings. It is possible that the focus group framing or even the nature of discussing these issues in a group setting may have led to reporting bias. There may also be other factors that did not emerge in the discussions. Due to the design of the study, for those participants recruited at sites other than HIV clinics, HIV status was my self-report and for all participants HCV status was self-reported. Further, we could not confirm self-reports of prior testing and it is therefore important to note that what patients reported were their perceptions and memories. Also, it is impossible to discern the extent to which HCV treatment was medically necessary for the HCV-positive focus group participants. The data collected through focus groups are qualitative and further quantitative survey data about the proportion of DUs having positive, neutral, or negative experiences would be valuable. Further, these focus groups were conducted during 2008–2009 and issues of awareness and access may have changed; however, the availability of improved therapies only increases the need to have clear understandings of potential barriers. Finally, due to funding limitations, 1) focus groups were not conducted with white drug users, which would have been useful for comparison; and 2) only English-speaking drug users were eligible for this study, and therefore our findings may not reflect those of non-English speaking drug users.

HCV remains a critical public health challenge among drug users, and the numbers of deaths due to HCV have surpassed those due to HIV/AIDS [42]. A recent meta-analysis has demonstrated that a sustained virologic response after treatment is associated with a reduced incidence of hepatocellular carcinoma underscoring the importance of engaging HCV infected patients in treatment [43]. HCV is a major cause of preventable morbidity and mortality among IDUs; scaled-up efforts to prevent HCV are imperative. Efforts to increase or establish HCV surveillance as well as the development of comprehensive and effective strategies to reduce transmission among IDUs are urgently needed. Public health approaches to HCV may benefit from expanding access to and awareness of voluntary HCV testing sites and treatment services. Standardized post-test counseling messages and active referral are critical in efforts to promote stronger linkages between HCV testing and care. Concrete, active referral linkages may also be needed [44]. Additionally, as with the Seek, Test, and Treat strategy being employed to reduce population HIV rates, programs targeted at increasing rates of HCV treatment among drug users might be an important strategy to reduce the number of HCV-positive persons, thus reducing both overall risk to individual drug users and reducing HCV prevalence at the population level.

## Competing interests

The authors declare that they have no competing interests.

## Authors’ contribution

DCP and CLM conceived of the study, and supervised all aspects of its conduct. DCP supervised the analysis and writing of the manuscript. AEJ assisted in the conduct of the focus groups, conducted the final coding and analyses, and wrote the first and subsequent manuscript drafts. CLM and PMG conducted the focus groups. PMG, CM, NP, KB, LG, EK, RMS, DCD, and JLS contributed to the study design, coding, analyses, and writing. All authors read and approved the final manuscript.
